# Recessive Charcot-Marie-Tooth and multiple sclerosis associated with a variant in *MCM3AP*

**DOI:** 10.1093/braincomms/fcz011

**Published:** 2019-09-03

**Authors:** Maryam Sedghi, Ali-Reza Moslemi, Macarena Cabrera-Serrano, Behnaz Ansari, Majid Ghasemi, Mojtaba Baktashian, Ali Fattahpour, Homa Tajsharghi

**Affiliations:** 1 Medical Genetics Laboratory, Alzahra University Hospital, Isfahan University of Medical Sciences, Isfahan, Iran; 2 Department of Pathology, Sahlgrenska University Hospital, Gothenburg University, Gothenburg, Sweden; 3 Department of Neurology, Hospital Universitario Virgen del Rocio, Sevilla, Spain; 4 Instituto de Biomedicina de Sevilla, Universidad de Sevilla, Sevilla, Spain; 5 Department of neurology, Isfahan University of Medical Sciences, Isfahan, Iran; 6 Radiology Resident, Department of Radiology, Mashhad University of Medical Sciences, Mashhad, Iran; 7 Division of Biomedicine, School of Health Science, University of Skovde, SE-541 28 Skovde, Sweden

**Keywords:** multiple sclerosis, Charcot-Marie-Tooth, neuropathy, *MCM3AP*, GANP

## Abstract

Variants in *MCM3AP*, encoding the germinal-centre associated nuclear protein, have been associated with progressive polyneuropathy with or without intellectual disability and ptosis in some cases, and with a complex phenotype with immunodeficiency, skin changes and myelodysplasia. *MCM3AP* encoded protein functions as an acetyltransferase that acetylates the replication protein, MCM3, and plays a key role in the regulation of DNA replication. In this study, we report a novel variant in *MCM3AP* (p.Ile954Thr), in a family including three affected individuals with characteristic features of Charcot-Marie-Tooth neuropathy and multiple sclerosis, an inflammatory condition of the central nervous system without known genetic cause. The affected individuals were homozygous for a missense *MCM3AP* variant, located at the Sac3 domain, which was predicted to affect conserved amino acid likely important for the function of the germinal-centre associated nuclear protein. Our data support further expansion of the clinical spectrum linked to *MCM3AP* variant and highlight that *MCM3AP* should be considered in patients with accompaniment of recessive motor axonal Charcot-Marie-Tooth neuropathy and multiple sclerosis.

## Teaser

Segregation of *MCM3AP* variants has previously been reported in patients with progressive polyneuropathy with or without intellectual disability. Sedghi *et al.* report the co-segregation of *MCM3AP* variant with recessive Charcot-Marie-Tooth and development multiple sclerosis in three affected individuals of a family, further expanding the clinical spectrum linked to *MCM3AP* variant.

## Graphical Abstract

**Graphical Abstract:**
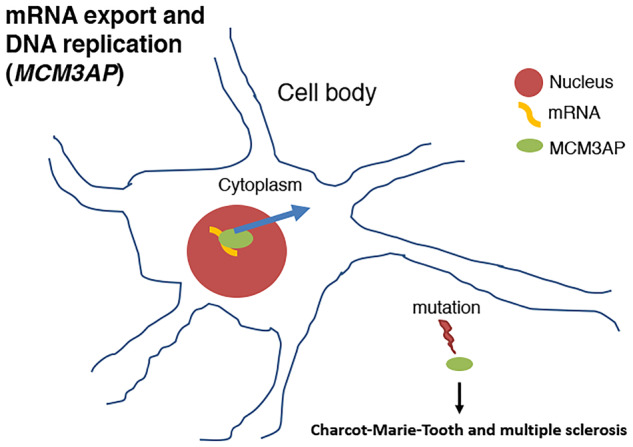

## Introduction

Charcot-Marie-Tooth (CMT) is an hereditary polyneuropathy with a wide genetic heterogeneity (Neuromuscular Disease Centre; http://neuromuscular.wustl.edu/time/hmsn.html) and varied Mendelian inheritance manner, including autosomal dominant, autosomal recessive and X-linked disorders. Affected individuals present with slowly progressive distal motor neuropathy resulting in muscle weakness and atrophy in the feet and or hands and the common feature of pes cavus foot deformity.

Very recently the first cases of recessive CMT hereditary neuropathy, caused by variants in *MCM3AP*, have been reported (Karakaya *et al.*, 2017; Ylikallio *et al.*, 2017; Kennerson *et al.*, 2018). Affected individuals presented with severe childhood onset primarily axonal or demyelinating CMT neuropathy with mild-to-moderate intellectual disability (Ylikallio *et al.*, 2017), or sensorymotor polyneuropathy and distal weakness, with or without mild intellectual disability, strabismus and/or ophthalmoparesis (Karakaya *et al.*, 2017). Variants in *MCM3AP* were initially linked to intellectual disability in an affected sibling pair with progressive polyneuropathy (Schuurs-Hoeijmakers *et al.*, 2013) and later with a complex phenotype with immunodeficiency, genomic instability, skin changes and myelodysplasia in a child (Gatz *et al.*, 2016).

The ubiquitously expressed *MCM3AP* (OMIM 603294) encodes the multi-domain germinal-centre associated nuclear protein (GANP), which functions as an mRNA export factor (Wickramasinghe *et al.*, 2010; Umlauf *et al.*, 2013). GANP is an alternative splice variant of *MCM3AP* with a carboxyl-domain that is shared with minichromosome maintenance complex component 3 associated protein (MCM3AP; Abe *et al.*, 2000; Wickramasinghe *et al.*, 2011). MCM3AP functions as an acetyltransferase that acetylates the replication protein MCM3 and plays an essential role in the translocation of MCM3 from the cytoplasm into the nuclei (Takei *et al.*, 2001) and in the regulation of DNA replication (Takei *et al.*, 2002).

Multiple sclerosis (MS) is an autoimmune neurological disease of the central nervous system (CNS; Calabresi, 2004), in which axons in the CNS being demyelinated to varying degrees (Weinshenker, 1996; Olek, 1999). The disease typically presents in adults 20–50 years of age (Calabresi, 2004; Goldenberg, 2012), although about 3–10% of all MS cases have their first manifestations in childhood or adolescence (Banwell *et al.*, 2007; Waldman *et al.*, 2014). MS is a chronic condition with widely variable symptoms and ultimately causing deficiency in sensation, movement, cognition or other functions depending on which areas of the brain or spinal cord are involved (Goldenberg, 2012; Thompson *et al.*, 2018). The early course of the disease includes weakness, tingling, numbness, blurred vision, muscle stiffness, thinking and emotional problems and urinary difficulties (Compston and Coles, 2008). Diagnosis of MS is based on the clinically compatible episodes of focal involvement of CNS with evidence of dissemination in time (at least two episodes, or new lesions identified in subsequent MRIs) and space (clinical relapses or radiological lesions involving different areas of the CNS; Thompson *et al.*, 2018). The presence of oligoclonal bands in cerebrospinal fluid supports the diagnosis (Thompson *et al.*, 2018). Although in a majority of cases, the cause is unknown, a combination of genetic susceptibility and environmental factors such as a viral infection appears to be involved in the development of MS (International Multiple Sclerosis Genetics Consortium *et al.*, 2007; Cree, 2014).

In this study, we report the co-segregation of a novel homozygous variant in *MCM3AP* in three affected individuals of a family with motor axonal CMT and normal intellectual ability who developed MS.

## Materials and methods

### Ethical approval

The study was approved by the ethical standards of the relevant institutional review board, the Ethics Review Committee in the Gothenburg Region (Dn1: 842-14). Informed consent was obtained from all parents included in this study after appropriate genetic counselling. Blood samples were obtained from patients, their parents and other available family members.

### Clinical evaluation

Medical history, physical examination and imaging were performed as part of routine clinical workup. For the three *MCM3AP* mutation-positive patients, extensive clinical follow-up was performed.

### Genetic analysis

Whole exome sequencing was performed on patients’ DNA. Bi-directional Sanger sequencing was performed in the patients and their unaffected parents and siblings. Detailed methods are provided in the Supplementary material.

## Data availability

The data that support the findings of this study are available from the corresponding author upon reasonable request.

## Results

### Clinical characteristics of patients

**Figure 1:**
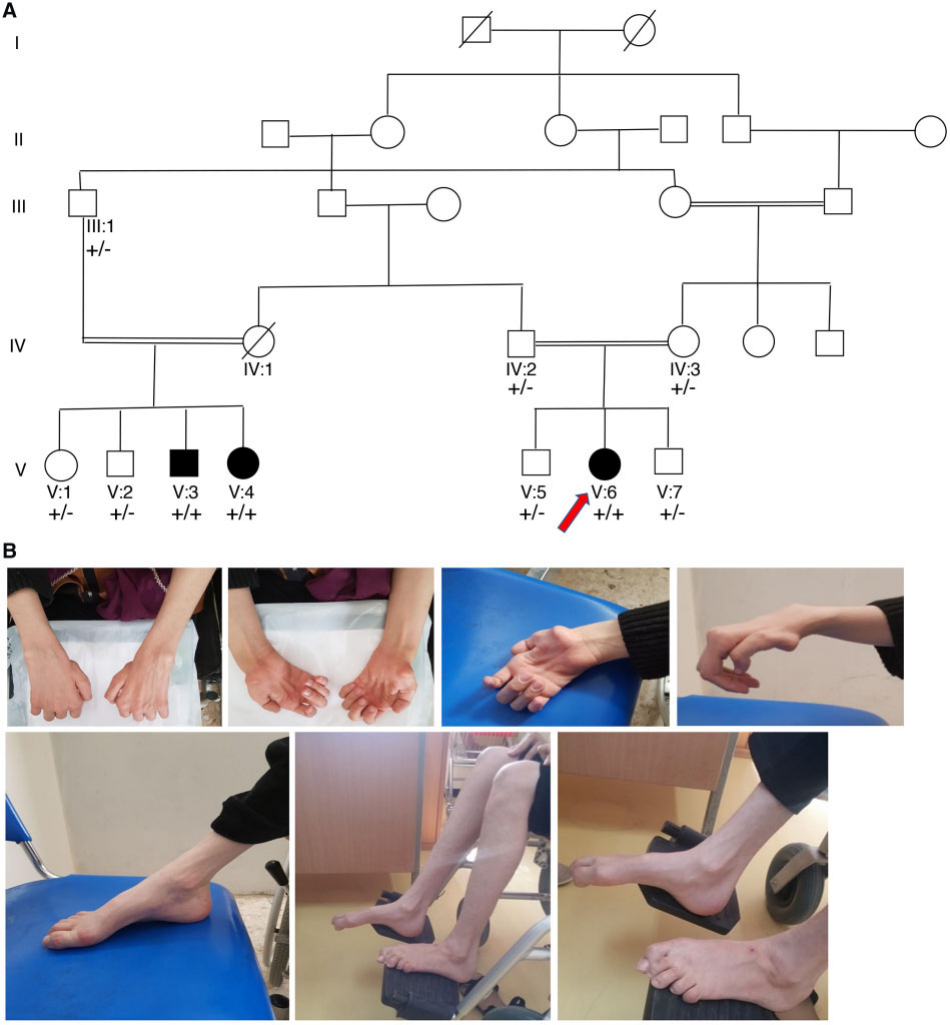
**Pedigrees of the family with *MCM3AP* variant and clinical features. Affected individuals are represented with shaded symbols.** Hands and feet deformities were shown in *Case V:6*. Wrist and finger drop, high arch and hammertoe deformity, are shown.

We report three family members (Cases V:6, V:3 and V:4) from a Caucasian family with consanguinity (Fig. 1). They presented with predominantly motor axonal CMT with an early onset and developed MS. Family history indicated no individual diagnosed with CMT or MS alone.

*The index case (V:* *6)*, a 30-year-old female, is the only affected child of healthy first cousin parents (Fig. 1). Pregnancy and delivery were uneventful. There was no history of prenatal or neonatal concerns. She was able to sit independently at 6 months of age and walk without support at 12 months of age. She was first examined at 4 years of age for tremor in hands. She was followed at 7 years of age for distal muscle weakness mainly at upper extremities. She learnt reading at 7 years of age and showed normal intellectual and cognitive development. At 12 years of age, she showed distal muscle atrophy and weakness of both upper and lower extremities, foot drop and finger contractures. She had impaired fine motor skills, with weakness and wasting of the intrinsic hand muscles, claw hands and writing difficulty (Fig. 1B). She had Achilles contracture. The sensory examination showed distal loss. Electrodiagnostic tests at age 12 revealed severe axonal motor neuropathy affecting both upper and lower extremities. Sensory nerve conduction studies were within normal range. Other features are listed in Table 1. At 19 years of age, she had a subacute presenting episode of right hemiparesis and blurred vision. These symptoms improved after treatment with prednisolone. But from age 20 she had recurrent episodes of diplopia, vertigo or gait difficulties.

**Table 1:** Clinical findings of Cases with homozygous *MCM3AP* mutation

	Case V:6		Case V:3		Case V:4	
Ethnicity	Iranian		Iranian		Iranian	
Sex	Female		Male		Female	
Age (year)	30		39		44	
Age at last examination (year)	30		39		44	
Start walking (age, months)	12		14		13	
Loss of ambulation (age)	23		39		She is ambulant	
Intellectual disability (+/−)	−		−		−	
Upper motor neuron sign (+/−)	−		−		−	
Deep tendon reflexes	Absent		Absent		Absent	
Motor developmental delay (+/−)	−		−		−	
Distal weakness and atrophy (upper or lower limbs) (+/−)	+		+		+	
Ankle dorsiflexion	Weak		Weak		Weak	
Finger/wrist drop (+/−)	+		+		+	
Increased tone (+/−)	−		−		−	
Tremor (+/−)	+		+		+	
Ataxia (+/−)	Wheelchair bound		Wheelchair bound		+	
Finger flexor contracture (+/−)	+		+		+	
Contracture in knees (+/−)	+		+		−	
Clubfeet (+/−)	−		−		−	
Hammertoes (+/−)	+		+		+	
Hearing loss (+/−)	−		−		−	
Babinski sign (+/−)	−		−		−	
Nerve conduction studies						
Sensory nerve conduction study	Normal		Normal		Normal	
Motor nerve conduction study	Reduced CMAPs		Reduced CMAPs		Reduced CMAPs	
Spinal MRI	Cord atrophy		Cord atrophy		No atrophy	
Brain MRI findings	Multiple plaque in peri and para ventricular space, juxta cortical, infra tentorial and corpus callous with enhancing plaque		Multiple plaque in peri and paraventricular space, juxta cortical, infra tentorial and corpus callous with one enhancing lesion and brain atrophy		Multiple plaque in para and peri ventricular, corpus callous, juxta cortical and infratentorial (pons) spaces	
Symptoms of MS (age, year)	20	30	34	39	40	44
Paresthesias (+/−)	+ (in back)	+ (in back)	−	−	−	−
Dysartheria (+/−)	−	−	−	+	−	+
Diplopia (+/−)	+	−	−	−	−	−
Blurred vision (+/−)	+	−	−	−	−	−
Vertigo (+/−)	+	−	−	−	−	−
Urinary incontinence (+/−)	+	+	−	+	−	+
Gait disturbances (+/−)	+	+ wheelchair bound	−	+ wheelchair bound	+	+
Other			Seizure, pseudobulbar affect		Pseudobulbar affect	
*MCM3AP* mutation	Homozygous p.Ile954Thr		Homozygous p.Ile954Thr		Homozygous p.Ile954Thr	

^+^Present; ^−^Absent.

CMAP = compound muscle action potential.

She lost ambulation at the age of 23 years and became wheelchair bound. At 30 years of age, muscle strength of the upper limbs, with Medical Research Council (MRC) grade, was 0/5 in finger extensor, finger abduction and thumb abduction, 2/5 in wrist extensor, −3/5 in wrist flexor and −4/5 in finger flexor, bilaterally. The muscle strength was +4/5 in biceps, triceps and deltoid muscles. She had bilateral foot drop with muscle strength 2/5 in foot dorsiflexion, eversion and inversion. The plantar flexion, knee flexion and extension was 4/5. She had reduced muscle tone (Fig. 1B). In the sensory examination, she showed glove and stocking sensory loss. In the cerebellar examination, alternate movement was normal but due to severe weakness, we could not exam the tandem gait, heel to shin and finger to nose.

**Figure 2:**
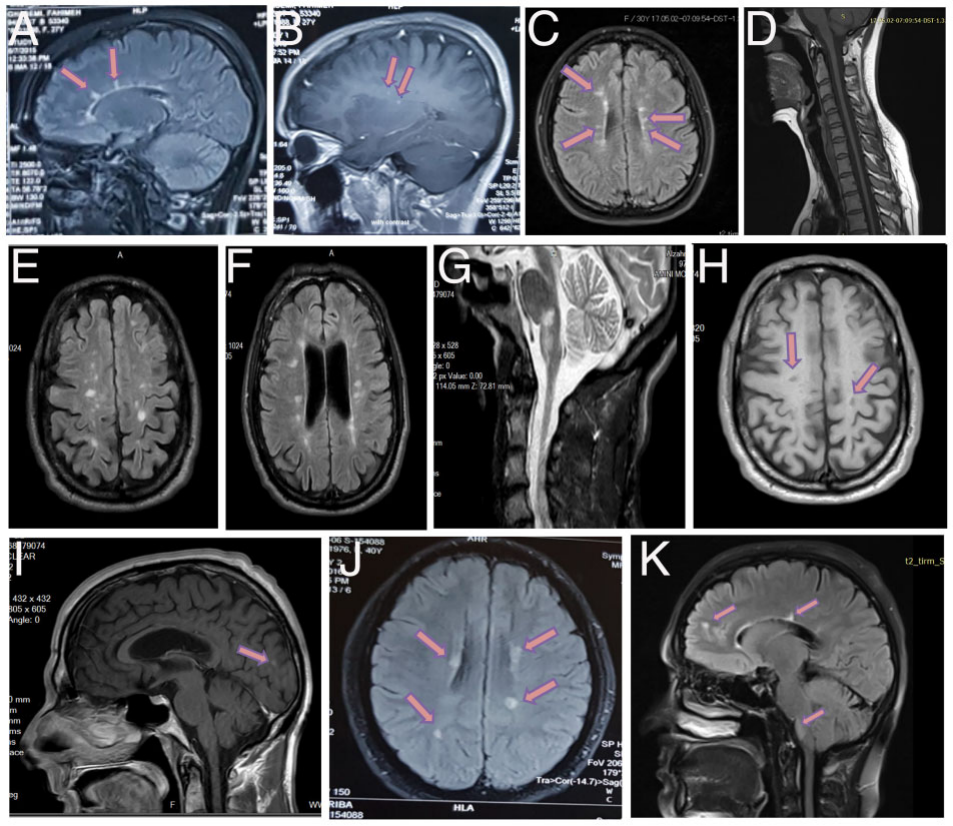
**Brain MRI.** Axial contrast-enhanced T2W MRI image of the brain of Cases V:6, V:1 and V:3. *Case V:6* (**A**) Midline sagittal flair (**B**) and parasagittal T1+GAD images, at 27 years of age, show multiple high-signal foci in the corpus callosum and paraventricular space with enhancing. (**C**) Axial FLAIR image, at 29 years of age, shows bilateral periventricular high-signal foci. (**D**) Spinal cord atrophy is shown. *Case V:3* (**E, F**) Axial FLAIR images, at the age of 39, show bilateral juxta cortical, periventricular and paraventricular high-signal foci and ventricular dilation due to brain atrophy. (**G**) Sagittal T2 weighted image shows confluent plaques in pons and medulla. (**H**) Axial T1 weighted image shows some black hole lesions in paraventricular space due to severe axonal injury and loss. (**I**) Faint enhancement after contrast media administration. *Case V:4* (**J**) At the age of 42, axial FLAIR image shows right periventricular, and juxta cortical and left paraventricular high-signal foci. (**K**) Sagittal Flair image, at 44 years of age, shows periventricular and white matter lesions and involvement of pons.

MRI of the brain at 27 years of age indicated multiple high-signal plaques in the corpus callosum (Fig. 2A) and multiple enhancing periventricular lesions (Fig. 2B), which confirmed the MS diagnosis. At follow-up at the age of 29 years, MRI of the brain indicated multiple new bilateral periventricular high-signal foci in Flair sequences and spinal cord atrophy (Fig. 2C and D).

Cerebrospinal fluid evaluation showed increased IgG index. Oligoclonal bands were, however, not detected.

*Case V:* *3*, a 39-year-old male, is the first affected offspring of first-cousin parents (Fig. 1). Pregnancy and delivery were uneventful. There was no history of prenatal or neonatal concerns. He was able to sit independently at 7 months of age and walk without support at 14 months of age. He had tremor in hands from childhood. At 16 years of age, he presented with muscle weakness at both the upper and lower extremities. Nerve conduction studies demonstrated a severe motor neuropathy with normal sensory conduction. At the age of 34, he had a first generalized, tonic–clonic seizure. He also developed slurred speech (dysarthria), ataxia and diplopia. At last clinical examination aged 39, he had reduced muscle tone and predominant distal muscle atrophy in upper and lower limbs and lost ambulation due to muscle weakness. Generalized absence of deep tendon reflexes was seen. Abdominal reflexes were normal. Pinprick and proprioceptive sensation were normal. The eye movement examination was normal. Muscle strength of upper limbs was 4/5 in proximal and 3/5 in distal muscles, bilaterally. In the lower extremities, muscle strength was 2/5 in dorsiflexion, +4/5 in plantar flexion and +4/5 in the proximal muscles.

A brain MRI at the age of 39 showed multiple white matter lesions in bilateral juxta cortical, periventricular and periventricular spaces and brain atrophy, in addition to confluent plaques in pons and medulla (Fig. 2E–G). Some black hole lesions were apparent in T1 sequences in periventricular regions (Fig. 2H). In addition, some of the MRI images showed faint enhancement after contrast administration (Fig. 2I).

All the clinical features of the patient at the last examination are listed in Table 1.

*Case V:* *4*, a 44-year-old female, and sibling of the V:3, is the second affected child of her parents. Pregnancy and delivery were uncomplicated and no concerns were noted in the prenatal or neonatal periods. At 7 months of age, she was able to sit independently, and she walked without support at 13 months of age. She had hand tremor from childhood. She has been followed since 13 years of age for tremor and distal muscle weakness predominantly in hands. At 16 years of age, electromyography was consistent with a motor axonal neuropathy with the preservation of sensory responses, similar to her younger brother (Case V:3). The clinical features are listed in Table 1.

At the age of 40, she developed a subacute episode of ataxia. At 42 years of age, she presented with dysarthria and diplopia. Clinical examination at 44 years of age showed reduced muscle tone and predominant distal muscle atrophy in both upper and lower limbs as well as generalized areflexia, sensory loss for pinprick, abnormal vibration sense in a glove and stocking distribution worse in her feet and positive Romberg sign. Power testing of the upper limbs disclosed weakness and atrophy of the small hand muscles and grip (finger extensor in left side: 2/5, in right side: 3/5, finger flexor in both sides: 4/5, finger abduction in left and right sides: 0/5, thumb abduction in both sides: 0/5, wrist flexor in both sides: 4/5, wrist extensor in both sides: 4/5. A better muscle strength was detected in proximal muscles: +4/5 in biceps, triceps and deltoid in both sides). In the lower extremities, muscle strength was 2/5 in dorsiflexion, 3/5 in plantar flexion and 4/5 in proximal muscles. Abnormalities of the cerebellar examination were noted; she could not walk correctly in Tandem gait and abnormality in heel to shin test was detected.

The MRI evaluation of brain at 42 years of age revealed multiple white matter plaques in periventricular, juxta cortical and left paraventricular areas (Fig. 2J), consistent with MS. Follow-up MRI at the age of 44 demonstrated juxta cortical, periventricular and paraventricular white matter lesions and involvement of pons (Fig. 2K). She has been treated with glatiramer acetate for the last year.

### Genetic findings

**Figure 3:**
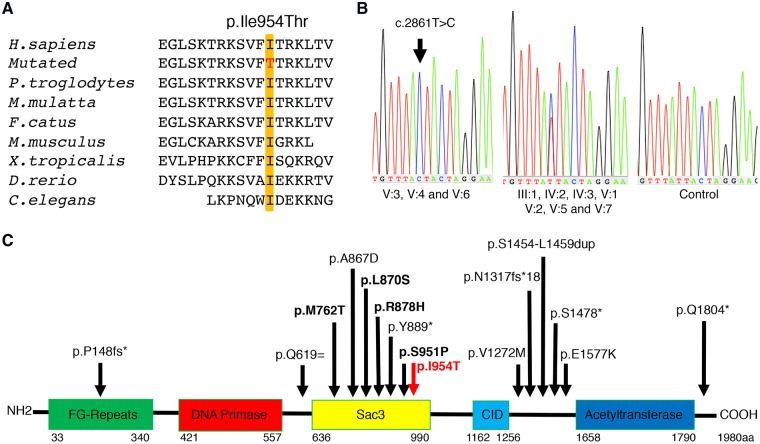
**Molecular genetics data.** (**A**) Multiple sequence alignment of the p.Ile954Thr region of the MCM3AP amino acid sequence confirms that the substitution affects an evolutionarily conserved residue (shaded). (**B**) Sanger sequence analysis demonstrates the presence of a novel homozygous missense mutation in exon 11 in *MCM3AP* (c.2861T>C) in Cases V:6, V:1 and V:3 (arrow). The apparently asymmetric parents and siblings were heterozygous for the variant. (**C**) The schematic illustration of GANP protein, consisting of different sub-domains, including the Sac3 domain. The previously reported variants of *MCM3AP* are shown with black arrows (bold indicates homozygous variants) and the novel p.Ile954Thr variant reported here is shown with red arrow. Adapted from Karakaya *et al.* (2017) by permission of Oxford University Press

Data from whole-exome sequencing on DNA from Cases V: 3 and V: 6 were analysed through the use of the Ingenuity Variant Analysis (IVA) software (Qiagen, Hilden Germany). The filtering strategy was initially concentrated on homozygous coding variants in known neurogenetic disease genes, selected based on variant databases Human Genome Mutation Database (HGMD) and ClinVar, and most recent literature (Bird, 1993; Didonna and Oksenberg, 2017). Only those changes that were predicted to be damaging or with unknown impact were analysed. An overview of the exome analysis is summarized in Supplementary Fig. 1A. All the rare homozygous missense/non-sense/frameshift variants identified in Cases V:3 and V:6 are listed in the Supplementary Table 1. A novel homozygous missense mutation in exon 11 of *MCM3AP* (NM_003906.4, c.2861T>C; Supplementary Fig. 1B), leading to substitution of a highly conserved isoleucine to threonine (p.Ile954Thr), was identified in Cases V:3 and V:6 (Fig. 3A). The p.Ile954Thr is not present in any public variant databases [Greater Middle East (GME) and Genome Aggregation Database (gnomAD; accessed February 2019)]. The p.Ile954Thr *MCM3AP* substitution is suggested to be disease causing by *in silico* predictors [MutationTaster, combined annotation dependent depletion (CADD; deleterious, scores 23.900], PolyPhen-2 function prediction (probably damaging), SIFT function prediction (deleterious) and PMut (pathogenic).

The *MCM3AP* variant was confirmed by PCR and bi-directional Sanger sequencing analysis, which also identified homozygosity for the c.2861T>C (p.Ile954Thr) in Case V:1, the affected sibling of Case V:3 (Fig. 3B). Co-segregation studies confirmed that the asymptomatic parents and the unaffected siblings in the family were heterozygous for the c.2861T>C variant (Figs 1A, 3B and Supplementary Fig. 1C).

The mutated residue, highly conserved among the orthologues, is located at the suppressor of actin 3 (Sac3) domain of the GANP (Fig. 3C), which is required for interaction with TREX-2 transcription-export complex (Jani *et al.*, 2012).

### Status of confirmed carriers

Co-segregation studies confirmed that all parents in the family were carriers of *MCM3AP* variant. All carrier parents and four unaffected siblings were examined by an experienced neurologist. They showed no evidence of neurological symptoms or signs.

## Discussion

In this study, we report the co-segregation of a variant in *MCM3AP* in three affected individuals of a family with recessive hereditary peripheral predominantly motor neuropathy, CMT, who developed clinical symptoms typical of MS. Multiples areas with demyelination in the brain and spinal cord were present in all three affected individuals, fulfilling the criteria for MS (Thompson *et al.*, 2018).

Although segregation of CMT with *MCM3AP* variants has previously been reported, the clinical features vary between the individuals in the present study and the previously reported. Unlike the previously reported cases with predominantly sensory motor CMT (Karakaya *et al.*, 2017; Ylikallio *et al.*, 2017), finding from electromyography from the individuals with *MCM3AP* p.Ile954Thr variant indicated a motor axonal CMT with normal sensory conduction studies, although later in the disease course two of the patients developed distal sensory loss. In addition, clinical findings including intellectual disability were not present in affected individuals in this family. Furthermore, the affected individuals developed clinical symptoms indicating CNS involvement and sequential brain and spinal cord imaging demonstrated multiple white matter lesions consistent with MS (Thompson *et al.*, 2018).

Concurrent central and peripheral demyelination has previously been reported (Amato and Barohn, 1996; Almsaddi *et al.*, 1998). Changes in CNS in patients with hereditary motor and sensory neuropathy due to rare mutations in *MPZ* (Watanabe *et al.*, 2002) or gene encoding peripheral myelin protein 22 (*PMP22*; Sanahuja *et al.*, 2005) have been identified. Furthermore, CNS involvement mimicking MS has been reported in X-linked CMT patients (Taylor *et al.*, 2003; Isoardo *et al.*, 2005; Zambelis *et al.*, 2008) and patient with history of CMT1A (Frasson *et al.*, 1997; Koros *et al.*, 2013). Nevertheless, concomitant central and peripheral demyelination represents a relatively uncommon clinical manifestation and changes in the CNS rarely fulfil the criteria for MS (Kilfoyle *et al.*, 2006; Koros *et al.*, 2013).

The co-segregation of *MCM3AP* variant with recessive motor axonal CMT and the development of MS in our patient is intriguing. In flies, it is suggested that GANP, with a vital role in the nuclear messenger RNA export in neurons, suppresses the TDP-43-mediated motor neuron degeneration (Sreedharan *et al.*, 2015). The location of the p.Ile954Thr variant at the Sac3 domain of GANP, which is required for interaction with TREX-2 components that links transcription with nuclear mRNA export (Jani *et al.*, 2012), may suggest an impact on nuclear mRNA export in neurons.

The underlying mechanism of MS is considered to be either destruction by the immune system or failure of the myelin production (Compston and Coles, 2008). A role of GANP in the immune response has been suggested and mice with GANP deficiency in immune cells show reduced affinity maturation of antibodies against T-cell-dependent antigens (Kuwahara *et al.*, 2004). In addition, a physiological role of GANP in B cell antibody maturation has been suggested (Singh *et al.*, 2013). B cells, in turn, have the potential to modulate the responses of other immune cells including T cells and myeloid cells, which suggests functions of B cells that may be relevant in both the peripheral and CNS diseases (Li *et al.*, 2018). Furthermore, the involvement of GANP in neoplasms originating in the CNS has been suggested (Ohta *et al.*, 2009).

Taken together, this may suggest that the co-segregation of *MCM3AP* with concurrent CMT and MS could be likely, although an incidental accompaniment of CMT and MS in this family could not be excluded. The pathologic mechanism of the p.Ile954Thr variant on the development of the lesions in the white matter of the CNS present in the patients remains, however, unknown and requires further investigations.

To the best of our knowledge, this is the first report on an autosomal recessive CMT and coexistent CNS changes consistent with MS, based on clinical manifestations and brain and spinal imaging. However, further families with variants in *MCM3AP* segregating with accompaniment of CMT and MS will be required to build on the association.

## Web resources

The following Databases were used in this study:

The Exome Variant Server: NHLBI Exome Sequencing Project (ESP), Seattle, WA;

URL: http://evs.gs.washington.edu/EVS/

1000 Genome Project Database: http://browser.1000genomes.org/index.html

Exome Aggregation Consortium (ExAC): http://exac.broadinstitute.org/

Human Background Variant DataBase: http://neotek.scilifelab.se/hbvdb/

Genome Aggregation Database (GnomAD): http://gnomad.broadinstitute.org/

ClinVar: http://www.ncbi.nlm.nih.gov/clinvar/

Human Gene Mutation Database: http://www.hgmd.cf.ac.uk/ac/index.php

Greater Middle East (GME) Variome web: http://igm.ucsd.edu/gme/index.php

Ensembl genome browser: http://www.ensembl.org/

MutationTaster: http://mutationtaster.org/

PMut: http://mmb.irbbarcelona.org/PMut

PolyPhen-2: http://genetics.bwh.harvard.edu/pph2/

SIFT: http://sift.bii.a-star.edu.sg/

## Consent to publish

The patients and family members in this study provided written informed consent to publish their family trees, and family data, including photographs.

## Supplementary Material

fcz011_Supplementary_Data

## Abbreviations

CNS: central nervous system
CMT: Charcot-Marie-Tooth
GANP: germinal-centre associated nuclear protein
MCM3AP: minichromosome maintenance complex component 3 associated protein
MRC: Medical Research Council
MRI: magnetic resonance imaging
MS: multiple sclerosis
