# Supplementary material for: Recessive Charcot-Marie-Tooth and multiple sclerosis associated with a variant in MCM3AP
Source: Brain Commun. 2019 Sep 3;1(1):fcz011. doi: 10.1093/braincomms/fcz011 (PMC7425404; doi:10.1093/braincomms/fcz011)
Supplement: fcz011_Supplementary_Data [file fcz011_supplementary_data.zip › Supplementary Table 1.pdf]

Supplementary Table 1. All the rare homozygous variants identified in both Cases V:3 and V:6.

Chr	Position	Gene region	Gene symbol	Transcript variant	Protein variant	dbSNP ID	gnomAD frequency % (gnomAD Homozygous count)
3	190572860	3'UTR	*GMNC*	c.*224A>G	-	1194675262	-
3	75779510	3'UTR	*ZNF717*	c.*254T>C, c.*91T>C	-	79347388	-
6	32547005	Intronic	*HLA-DRB1*	c.788_124A>T	-	33962457	-
6	32548178	Intronic	*HLA-DRB1*	c.764_131T>C	-	9269755	-
6	32548722	Intronic	*HLA-DRB1*	c.653_89G>T	-	9269767	-
6	32548804	Intronic	*HLA-DRB1*	c.653_171G>C	-	9269775	-
6	32548852	Intronic	*HLA-DRB1*	c.653_219G>T	-	9269776	-
6	32548853	Intronic	*HLA-DRB1*	c.653_220T>C	-	9269777	-
6	32548896	Intronic	*HLA-DRB1*	c.653_263T>C	-	9269778	-
6	32549711	Intronic	*HLA-DRB1*	c.371_96C>T	-	9269814	-
11	89701958	Exonic	***TRIM64/TRIMB***	c.287A>C	**p.E96A**	201411460[1]	0.448 (13)
12	133633459	Intronic	*ZNF84*	c.*47-81A>G, c.239-6206A>G, c.239-81A>G, c.239-84A>G	-	608270	-
12	133634450	Exonic, Intronic	*ZNF84*	c.1146A>C, c.1149A>C, c.239-5215A>C	-	623100	-
12	133639605	3'UTR, Intronic	*ZNF84*	c.*4087T>G, c.239-60T>G	-	13678	-
19	55258650	Intronic	*CTB_61M7.1, KIR2DL3*	c.665-137C>T, n.35+22615C>T	-	75102618	0.015 (0)
21	43897478	Exonic	***RSPH1***	c.536T>G/c.650T>G	**p.L179W/ p.L217W**	138007679[2]	0.725 (11)
21	44839263	Exonic	***SIK1/SIK1B***	c.1215G>A	**p.M405I**	34987632[3]	0.384 (5)
21	47320422	Intronic	***PCBP3***	c.190-83G>A, c.94-83G>A, n.468-83G>A,	**-**	554504547	0.003 (0)

				n.658-83G>A			
21	47686009	Exonic	***MCM3AP***	c.2861T>C	**p.I954T**	-	0
22	17173012	Intronic, ncRNA	*KB_7G28*	n.2538G>A, n.492-5374C>T, n.89G>A	-	2845360	-
22	17175554	Intronic, ncRNA	*KB_7G28*	n.-16C>T, n.2434C>T, n.492-2832G>A	-	2845363	0
22	17178204	Intronic, ncRNA	*TPTEP1*	n.1582C>T, n.492_182G>A	-	2845370	0.005 (0)
22	17182168	ncRNA	*VWFP1*	n.479C>T	-	4008538	-
22	17182236	Intronic	*VWFP1*	n.445-34G>T	-	4008539	-
22	17182281	Intronic	*VWFP1*	n.445-79C>T	-	2906869	-
22	17182282	Intronic	*VWFP1*	n.445-80G>C	-	2906870	-
22	24343454	Exonic, Intronic	*GSTT4*	c.266T>C *, n.206-1009T>C	p.P89P	140292[4]	0.919 (2)

Abbreviations: Chr: Chromosome

Variants predicted to be deleterious are indicated in bold.

Clinical significance reported in ClinVar (Submitted interpretations and evidence):

[1]Not reported in ClinVar.

[2]The variant Leu179Trp/Leu217Trp of *RSPH1* is not expected to have clinical significance because it has been identified in 1.0% (87/8600) of European American chromosomes from a broad population by the NHLBI Exome Sequencing Project (http://evs.gs.washington.edu/EVS; dbSNP rs138007679).

[3]Lines of evidence used in support of classification of Met405Ile as benign. General population or subpopulation frequency is too high to be a pathogenic mutation based on disease/syndrome prevalence and penetrance

[4]Not reported in ClinVar.
