# Supplementary material for: Recessive Charcot-Marie-Tooth and multiple sclerosis associated with a variant in MCM3AP
Source: Brain Commun. 2019 Sep 3;1(1):fcz011. doi: 10.1093/braincomms/fcz011 (PMC7425404; doi:10.1093/braincomms/fcz011)
Supplement: fcz011_Supplementary_Data [file fcz011_supplementary_data.zip › Supplementary data.pdf]

Supplementary information

Content

1. **Supplementary Methods**
2. **Supplementary References**

1. **Supplementary Methods**

Brain Magnetic resonance imaging (MRI) evaluation

MRI examination of the brain is performed using the T1 weighted axial, T2 weighted sagittal, coronal, axial, FLAIR axial, sagittal proton, axial sequences with and without contrast media.

DNA isolation

Extraction of genomic DNA was performed from whole blood from probands and family members, using DNeasy Blood & Tissue kit (Qiagen, Hilden Germany), according to the manufacturer's instructions.

Genetic analysis

Next Generation sequencing

Whole exome sequencing (WES)

WES was performed on DNA from Cases V:3 and V:6, as previously described [1]. Briefly, target enrichment was performed with 3 μg genomic DNA using the SureSelectXT Human All Exon Kit version 5 (Agilent Technologies, Santa Clara, CA, USA) to generate barcoded whole-exome sequencing libraries. Libraries were sequenced on the HiSeq2000 platform (Illumina, San Diego, CA, USA) as paired-end 2×150 bp reads with 60x target coverage. Quality assessment of the sequence reads was performed by generating QC statistics with FastQC

(http://www.bioinformatics.bbsrc.ac.uk/projects/fastqc). Read alignment to the reference human genome (hg19, UCSC assembly, February 2009) was done using BWA [2] with default parameters. After removal of PCR duplicates (Picard tools, http://picard.sourceforge.net) and file conversion (SAMtools) [3], quality score recalibration, indel realignment and variant calling were performed with the HaplotypeCaller algorithm in the GATK package [4] based on established best practices [5].

WES variant annotation, filtering and prioritisation

Variants were annotated with ANNOVAR [6] using a wide range of databases such as dbSNP build 135, dbNSFP, KEGG, the Gene Ontology project and tracks from the UCSC. A filtering strategy, directed to disease gene candidates, was performed by QIAGEN's Ingenuity® Variant Analysis™ software (www.qiagen.com/ingenuity). Ingenuity Variant Analysis combines analytical tools which annotates variants and displays data including Polyphen, SIFT and CADD scores. We focused initially on coding variants in known neurogenesis disease genes, e.g. where the mutation produced a missense change, stop gain or stop loss, frameshift or essential splicing change. Given the known consanguinity in the family, the initial filtering strategy was also focused on homozygous variants. Only those changes that were predicted to be damaging (disease-associated according to computed American College of Medical Genetics and Genomics (ACMG) guidelines classification: pathogenic and likely pathogenic or with uncertain significance) were analysed. We excluded variants that were frequent in control datasets (>1% in dbSNP [7], the Exome Variant Server (EVS) (NHLBI) (http://evs.gs.washington.edu/EVS/), the gnomAD (http://gnomad.broadinstitute.org/), and the Human Background Variant Database (http://neotek.scilifelab.se/hbvdb/).

Polymerase chain reaction (PCR) and Sanger sequencing

PCR and bi-directional Sanger sequencing of *MCM3AP* was performed on proband and relatives. Primer details and conditions are available upon request.

2. Supplementary References

1. Kariminejad A, Ghaderi-Sohi S, Hossein-Nejad Nedai H et al. Lethal multiple pterygium syndrome, the extreme end of the RYR1 spectrum. BMC Musculoskelet Disord 2016; 17: 109.
2. Li H, Durbin R. Fast and accurate short read alignment with Burrows-Wheeler transform. Bioinformatics 2009; 25: 1754-1760.
3. Li H, Handsaker B, Wysoker A et al. The Sequence Alignment/Map format and SAMtools. Bioinformatics 2009; 25: 2078-2079.
4. McKenna A, Hanna M, Banks E et al. The Genome Analysis Toolkit: a MapReduce framework for analyzing next-generation DNA sequencing data. Genome Res 2010; 20: 1297-1303.
5. DePristo MA, Banks E, Poplin R et al. A framework for variation discovery and genotyping using next-generation DNA sequencing data. Nat Genet 2011; 43: 491-498.
6. Wang K, Li M, Hakonarson H. ANNOVAR: functional annotation of genetic variants from high-throughput sequencing data. Nucleic Acids Res 2010; 38: e164.
7. Sherry ST, Ward MH, Kholodov M et al. dbSNP: the NCBI database of genetic variation. Nucleic Acids Res 2001; 29: 308-311.
