# Supplementary material for: Recessive Charcot-Marie-Tooth and multiple sclerosis associated with a variant in MCM3AP
Source: Brain Commun. 2019 Sep 3;1(1):fcz011. doi: 10.1093/braincomms/fcz011 (PMC7425404; doi:10.1093/braincomms/fcz011)
Supplement: fcz011_Supplementary_Data [file fcz011_supplementary_data.zip › Supplementary Figure 1.pdf]

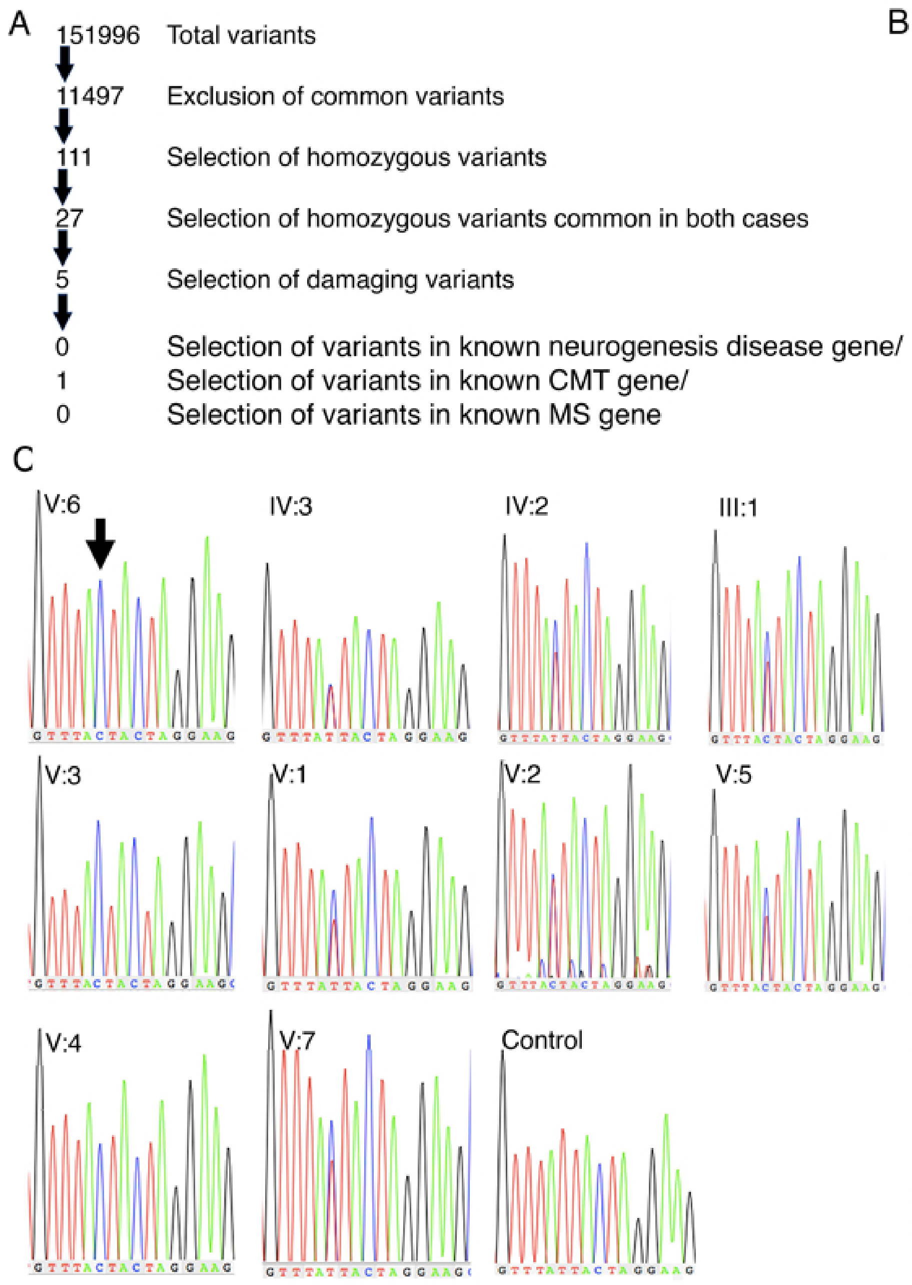

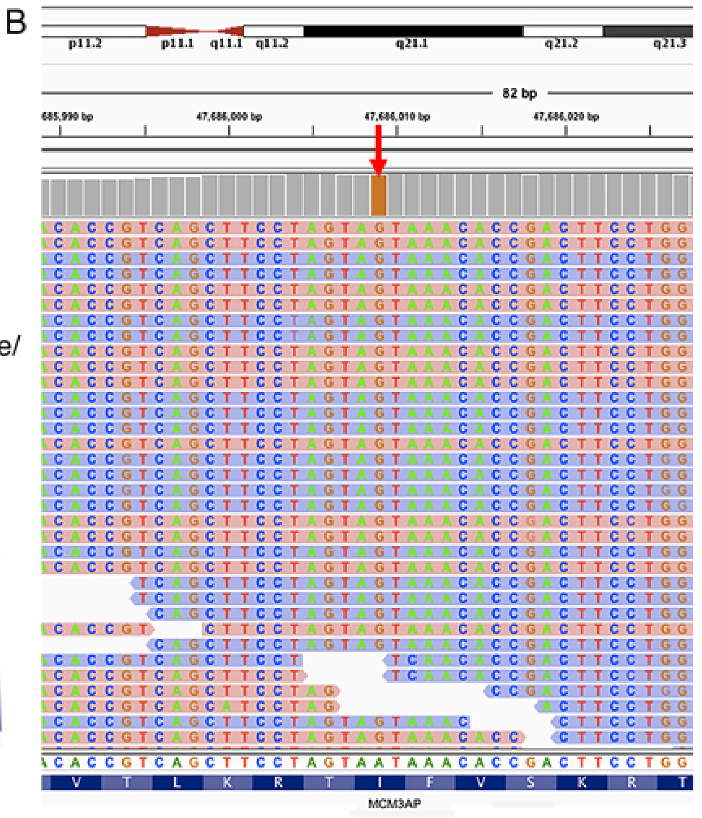

Supplementary Figure 1. Molecular genetics analysis and data. (A) Overview of the exome analysis of Cases V:3 and V:6. Altogether 151996 single nucleotide variants (SNVs) were identified as a result of variant calling in both Cases V:3 and V:6. Of these, variants that were not listed, or were present in less than 1% frequency in the gnomAD, 1000 Genomes Project, ExAC and NHLBI ESP exomes databases were filtered, resulting in 11497 SNVs. Of these, a total of 111 variants were homozygous in both patients and 27 homozygous variants were common in both Cases V:3 and V:6). Only 5 variants were predicted to be deleterious. Selection of variants in known CMT gene indicated MCM3AP p.I954T as the only deleterious variants. Selection of variants in known neurogenesis disease and MS genes indicated no variant. (B) Whole exome sequence analysis demonstrates the presence of homozygous mutations in MCM3AP in the patients (red arrow). (C) Sanger sequence analysis demonstrates the presence of a novel homozygous missense mutation in exon 11 in MCM3AP (c.2861T>C) (arrow) in Cases V:6, V:1 and V:3. The asymmetric parents and siblings were heterozygous for the variant.
